# Transvaginal sonography and surgical findings in the diagnosis of endometriosis individuals: A cross-sectional study

**DOI:** 10.18502/ijrm.v21i6.13634

**Published:** 2023-07-24

**Authors:** Roya Padmehr, Khadijeh Shadjoo, Arash Mohazzab, Atefeh Gorgin, Roxana Karegar, Parvin Jaberipour, Zahra Sehat, Narges Maleki

**Affiliations:** ^1^Reproductive Biotechnology Research Center, Avicenna Research Institute, ACECR, Tehran, Iran.; ^2^Avicenna Infertility Clinic, Avicenna Research Institute, ACECR, Tehran, Iran.

**Keywords:** Endometriosis, Laparoscopy, Pathology.

## Abstract

**Background:**

Endometriosis is a challenging gynecological disease and a debilitating condition that profoundly affects the individual's quality of life. Besides pathological confirmation, diagnostic laparoscopy has been internationally accepted as the standard method to identify the accurate mapping of endometriosis. Transvaginal sonography (TVS) is the first non-invasive imaging modality to estimate the severity of endometriosis.

**Objective:**

This study aimed to evaluate the accuracy of TVS in affected women compared with surgical findings.

**Materials and Methods:**

This retrospective cross-sectional study surveyed 170 women with deep infiltrating endometriosis (DIE) referred to the endometriosis part of the Avicenna Infertility Center, Tehran, Iran and they underwent TVS followed by laparoscopy. Recorded data of individuals under study in the medical database system were reviewed. Finally, the agreement rate was calculated for ultrasound reports and intraoperative (IO) findings regarding ovarian endometrium, ovarian adhesion, involvement of cul-de-sac, rectovaginal septum, and bowel and ureter.

**Results:**

170 women with DIE entered the study. The agreement of TVS and IO findings were 86.76% for left ovarian endometriosis and 70.86% for right ovarian endometriosis, 93.90% for left ovarian adhesion, and 88.90% for right ovarian adhesion, 88.90% for a cul-de-sac, and 84.82% for bowel nodules. The findings, based on a laparoscopic assessment of the pelvic floor, were completely compatible with ultrasound reports (100%).

**Conclusion:**

TVS allows a preoperative evaluation in planning the surgical policy associated. TVS is beneficial for dedicated mapping of DIE; thus, an expert radiologist can aid the surgeon in preoperative evaluation and IO management.

## 1. Introduction

Endometriosis is a challenging gynecological disease and a debilitating condition that profoundly affects individuals' quality of life. It is estimated that endometriosis affects 10% of reproductive-age women; this effect increases to 35-50% in symptomatic individuals (1-3). Pelvis endometriosis has 3 main entities: peritoneal, ovarian, and deep infiltrating endometriosis (DIE) (4).

“DIE is the presence of endometriosis implants that penetrate the retroperitoneal space for a distance of 5 mm or more (3-5). DIE can involve the Douglas pouch, the rectovaginal septum, the intestine, the anterior pouch, and the uterosacral ligaments. Assessment of this disease is difficult only by physical examination (4-7). Transvaginal sonography (TVS) is currently considered a fundamental non-invasive diagnostic method to evaluate the extent of DIE within the pelvis and facilitate the choice of a safe and adequate surgical or medical treatment (7-9)”. The most common presentations include pelvic pain, infertility, dysmenorrhea, dyschezia, and urinary manifestations. However, the non-specific symptoms of DIE often result in a missed diagnosis or delayed approach (1). DIE mostly appears as a multifocal disease in some locations with a higher likelihood of involvement, such as the bowel, vagina, urinary system, etc. (2).

An imaging modality to diagnose endometriosis is decisive in the management of such a debilitating condition. TVS is one of the first methods of approaching women with pelvic pain and other abovementioned complaints. It also provides clinicians with important data about the location and extent of endometriosis; however, there have been several reports about potential differences between TVS results and surgical findings, especially for DIE (1, 3).

Yet no diagnostic imaging method has been introduced as the gold standard for endometriosis; diagnostic laparoscopy, besides pathological confirmation, has been internationally accepted as the standard method to identify the accurate mapping of endometriosis. Though it is invasive and completely based on the surgeon's skill and experience, it does not provide the ability for preoperative planning (4). Additionally, laparoscopy has its limitations in cases of pelvic deep infiltrating or extra-pelvic endometriosis (1).

Despite laparoscopic evaluation being the reference for diagnosing DIE, the findings may lead to overdiagnosis or even underestimation of lesions that may be faded and obscured based on their location (5). TVS is a cost-effective method compared to magnetic resonance imaging in the diagnosis of endometriosis specifics (6). It is often considered the first non-invasive imaging modality to estimate the severity of endometriosis (8, 9).

This research aims to evaluate the accuracy of pre-surgical TVS in women with endometriosis and to compare the reports with surgical findings reports.

## 2. Materials and Methods

This cross-sectional study investigated 170 women with DIE referred to an Endometriosis Center of Avicenna Infertility Center in Tehran, Iran and they underwent TVS following a laparoscopic approach from March 2019 to March 2021. The study aimed to evaluate the diagnostic accuracy of TVS in endometriosis individuals compared with surgical findings.

All laparoscopically confirmed endometriosis individuals were included in the study during the study period. We reviewed the recorded data of individuals in the medical database system of Avicenna Research Center, Tehran, Iran and all study variables, including clinical presentations (dysmenorrhea, chronic pelvic pain, dyspareunia, dyschezia, infertility, rectal bleeding during menstruation), sonography report, intraoperative (IO) mapping (in terms of ovarian endometrioma [OMA], ovarian adhesion, cul-de-sac involvement, bowel, and ureter nodules), and pathological reports were extracted from the database. To avoid measurement bias, all TVS were performed by 3 radiologists who were experts in the gynecological field and had access to the clinical data of women at the time of TVS. We put aside all exterior ultrasound reports, and in the case of previously assessed individuals, reassessment was done to make the results more homogenous.

All individuals underwent diagnostic and excisional laparoscopic surgery in Moheb-e-Kosar hospital, Tehran, Iran by highly experienced gynecological surgeons. The surgeons were entirely informed about pre-surgical TVS reports. The accurate mapping of the endometriosis was evaluated and recorded for each individual during surgery. Afterward, surgeons re-evaluated their mapping based on recorded videotapes of the surgery to determine if any uncertainty about the mapping existed. All tissue specimens were studied in the same pathological laboratory following a laparoscopy.

To assess the diagnostic agreement between surgery and ultrasound, we categorized the mapping results of both methods identically: 3-layer categorization for ovarian cysts (no cyst, non-endometriotic cyst, OMA) and cul de sac (not involved, partially obliterated, and completely obliterated) and dichotomous categorization (involved, not involved) for ovarian adhesion, tubes, ureter, and bowel.

### Ethical considerations

This study was approved by the Ethical Committee of Avicenna Research Institute, ACECR, Tehran, Iran (Code: IR.ACECR.AVICENNA.REC.1398.004). All participants have completed the written consent form.

### Statistical analysis

Statistical analysis was done using Stata software version 22. Continuous variables were reported as mean ± SD, and all categorical variables were shown as n (%). To calculate agreement measures, cross-tabulation was done between TVS reports and surgical findings, then Cohn'es Kappa coefficient (K) was calculated for variables without skewness, Berman's Kappa for those with skewness, and Weighted Kappa for multi-rank variables. In the case of dichotomous classification of diagnosis, sensitivity, specificity, positive predictive value, and negative predictive value were calculated. P-value < 0.05 was considered statistically significant.

## 3. Results

204 women enrolled in this study and 170 of them had DIE. They were aged between 19 and 49 yr (mean = 34.43) and had a body mass index of 17.42- 35.16 (mean = 25.29).

59 (28.9%) of them had primary infertility and 29 (14.2%) had secondary infertility. All women in this study underwent laparoscopic surgery after performing TVS. The definite diagnosis of endometriosis was confirmed for each individual's pathological study of specimens.

Based on surgical findings, among 170 cases, 146 had left OMA, and 130 had right OMA. 98% of individuals with left OMA had ovarian adhesion, whereas 95.5% for the right side. The agreement of TVS and IO findings were 86.76% (K = 0.60) for left OMA and 70.86% (K = 0.47) for right OMA. Table I shows the result of the ultrasound and surgery of the left and right ovaries. The agreement of ultrasound and laparoscopic findings for ovarian adhesion was 93.9% (K = 0.47) for the left side and 88.9% (K = 0.36) for the right side. To calculate the percentage of agreement, cases of cysts and endometrioma were considered.

Table II shows the result of the ultrasound of adhesion ovaries and surgery of ovaries. 167 individuals had cul-de-sac endometriosis. 97% had complete obliteration and 3% were partially obliterated. The rest of the cases had no cul-de-sac involvement. The agreement was 88.9% (K = 0.24) with TVS. Table III shows the results of the ultrasound and surgery of the cul-de-sac.

The findings during laparoscopy in terms of assessment of rectovaginal septum were divided into 3 groups: 1-no involvement, 2-presence of endometriosis, and 3-individuals. Having a thick pelvic floor the findings, based on the laparoscopic assessment of the pelvic floor was completely compatible with ultrasound reports (100%). Also, 34 individuals had positive, 27 individuals had no, and 2 individuals had thick pelvic floor involvement in ultrasound and laparoscopic assessment. 103 individuals had bowel nodules. The ultrasound was positive in 88.34% in terms of bowel involvement. Table IV shows theultrasound and surgery of the bowel with.84.82% agreement (K = 0.53).

Table II shows the laparoscopic findings of ureters. As it is obvious, the agreement between surgical findings and ultrasound were not considered in terms of ureters' involvement.

Table III shows the agreement between ultrasound reports and laparoscopic findings of fallopian tube involvement. As shown, the agreement between these 2 variables is not significant. According to table V, more agreement between TVS and surgery was found in left ovarian adhesion, followed by the left and right ureter. Also, the least agreement between TVS and surgery was in the right tube adhesion and right OMA.

As a summary, the agreement of TVS and IO were 86.76% for left OMA and 70.86% for right OMA, 93.90% for left ovarian adhesion and 88.90% for right ovarian adhesion, 88.90% for cul-de-sac obliteration, and 84.82% for bowel nodules. The findings, based on the laparoscopic assessment of the pelvic floor were completely compatible with the ultrasound reports (100%).

**Table 1:** Result of ultrasound and surgery of left and right ovaries


		**Surgery of ovary**					
**Ultrasound of ovary**		**Left ovary ${}^{\text{a}}$ **			**Right ovary ${}^{\text{b}}$ **		
		**No cyst**	**Non-endometriotic cyst**	**Endometrioma**	**No cyst**	**Non-endometriotic cyst**	**Endometrioma**
**Cyst**							
	**No cyst**	13 (46.4)	3 (10.7)	12 (42.8)	12 (26.6)	10 (22.2)	23 (51.1)
	**Non-endometriotic** **cyst**	1 (6.25)	1 (6.25)	14 (87.5)	5 (29.4)	3 (17.6)	9 (53)
**Endometrioma**		6 (4.8)	0 (0)	120 (95.2)	2 (1.94)	3 (2.91)	98 (95)
Data presented as n (%). Cohn'es Kappa test. ${}^{\text{a}}$ Agreement = 86.76%, Kohen = 56.76, Brennan = 0.60, ${}^{\text{b}}$ Agreement = 70.86%, Kohen = 0.47, Brennan = 0.42							

**Table 2:** Result of ultrasound of adhesion ovaries and surgery of ovaries


	**Adhesion surgery of ovaries**			
	**Left ovary ${}^{\text{a}}$ **		**Right ovary ${}^{\text{b}}$ **	
**Ultrasound of ovary**	**No**	**Yes**	**No**	**Yes**
	**No**	1 (11.1)	8 (88.9)	2 (15.4)	11 (84.6)
	**Yes**	1 (0.7)	135 (99.3)	4 (3.3)	118 (96.7)
Data presented as n (%). Cohn'es Kappa test. ${}^{\text{a}}$ Agreement = 93.9%, Brennan = 0.87, Kohen = 0.47, ${}^{\text{b}}$ Agreement = 88.9%, Brennan = 0.77, Kohen = 0.36				

**Table 3:** Result of ultrasound of and surgery of cull-de-sac in individuals of study


	**Surgery of cul-de-sac**		
**Ultrasound**	**No involvement**	**Partially obliterated**	**Complete obliteration**
	**No involvement**	1 (7.4)	0	12 (92.3)
**Partially obliterated**	1 (2)	2 (4)	47 (94)
**Complete obliteration**	0	3 (3)	103 (97)
Data presented as n (%). Cohn'es Kappa test. Agreement = 88.9%, Brennan = 0.56, Kohen = 0.24			

**Table 4:** Result of ultrasound and surgery of the bowel and ureter and tabulation


		**Surgery of ureter**				**Surgery of bowel**		**Surgery of tabulation**			
**Ultrasound**		<**Left ureter**		<**Right ureter**				**Left tube**		<**Right tube**	
		**No**	**Yes**	**No**	**Yes**	**No**	**Yes**	**No**	**Yes**	**No**	**Yes**
	**No**	172 (93)	13 (7)	163 (92.6)	13 (7.4)	4 (25)	12 (75)	96 (55.5)	77 (44.5)	103 (57.4)	76 (42.6)
	Yes ${}^{*}$	1 (50)	1 (50)	2 (75)	1 (25)	5 (5.2)	91 (94.8)	5 (16)	26 (84)	9 (36)	16 (64)
**Cohn'es Kappa**		<Agreement = 92.5% Brennan = 0.77 Kohen = 0.42		<Agreement = 91.6% Brennan = 0.68 Kohen = 0.38		<Agreement = 84.82 Brennan = 0.69 Kohen = 0.53		<Agreement = 87.5% Brennan = 0.75 Kohen = 0.42		<Agreement = 72% Brennan = 0.44 Kohen = 0.33	
Data presented as n (%). In tabulation, this was Hydro salpinx											

**Table 5:** The result of the kappa factor of ultrasound and surgery


	**Ovarian**							**Ureter involvement**		**Tube adhesion**	
**Compartment**	**Endometrioma**		**Adhesion**		**Cyst**		**Cul-de-**
**sac**	**Bowel**						
**involvement**					
	**Left**	**Right**	**Left**	**Right**	**Left**	**Right**		**Left**	**Right**	**Left**	**Right**
**Percentage of** **agreement**	86.7	70.86	93.9	88.9		88.9	84.8	92.5	91.6	87.5	72
**Brennan Kappa** **coefficient**	0.6	0.42	0.87	0.77		0.56	0.69	0.77	0.6	0.75	0.44
**Sensitivity**	82.19	75.38	94.4	91.4	0.9	77.39	92.80	88.34	7.1	7.1	25.2	17.3
**Specificity**	75	88.5	0.5	0.33	0.65	63.15	0.5	44.44	99.42	98.87	95.05	91.96
**PPV**	95.23	95.1	99.2	96.7	95.07	94.16	96.1	94.8	50	33.33	83.8	64
**NPV**	40.09	48.3	11.11	15.38	46.04	26.6	7.5	25	92.97	92.61	55.49	57.54
PPV: Positive predictive value, NPV: Negative predictive value												

## 4. Discussion

In this study, the agreement of TVS and IO were 86.76% for left OMA and 70.86% for right OMA, 93.90% for left ovarian adhesion and 88.90% for right ovarian adhesion, 88.90% for cul-de-sac obliteration, and 84.82% for bowel nodules. The findings, based on the laparoscopic assessment of the pelvic floor were completely compatible with ultrasound reports (100%). According to the results of our study and based on the percentage of agreement of TVS and IO findings in different anatomical regions, the most compatible results were obtained for the pelvic floor, ovarian adhesions, Douglas pouch, bowel, and OMA, respectively. However, our research demonstrates that the preoperative TVS evaluation may miss some lesions in specific regions, such as ureters and fallopian tubes.

In a study, it was concluded that TVS could provide a precise mapping of DIE, especially for the rectovaginal septum, vagina, rectum, bladder, and ureter. The results of their research were compatible with ours except for the ureters. The agreement percentages of ureters' involvement in our study were not considered, and we concluded that endometriosis of the ureters is not precisely locatable by preoperative TVS (1).

Additionally, the study concluded that TVS appears to be a reliable modality for the preoperative assessment of ureteral endometriosis. However, they have mentioned that the TVS results are more compatible with IO findings in individuals with larger size involvement, which leads to ureteral dilatation or hydronephrosis. Based on their results, a ureteric diameter ≥ 6 mm with a median diameter of 6.9 mm (range: 6-18 mm) had been confirmed during operation for all cases, and diameters less than that had not been reported in TVS (10, 11).

These 2 studies were both prospective, which may be responsible for the contrast with our results. Another reason could be related to the size of the endometriotic lesions and their anatomical region. The pelvic segment of ureters is more detectable by TVS by moving the probe from the midline toward the pelvic sides, which appears as a tiny, long tubular hypoechoic structure, and a thick hyperechoic wall. The ureters' endometriosis is detectable as nodule's which are hypoechoic lesions appearing around the course of the ureters and those ≥ 17 mm should particularly raise suspicion for ureteral endometriosis. However, the same technique is less applicable to the abdominal segment of the ureter (12, 13).

TVS is an operator-dependent imaging technique, and the type of study (retrospective versus prospective) is also very important. Moreover, due to the differences between probes, operators' experiences, and the anatomic region of ureter involvement (pelvic segment or abdominal segment), the differences between the results of our study and the abovementioned research are justifiable and more studies with larger sample sizes are needed. According to our study, the high accuracy of TVS in the detection of DIE lesions of the bowel, ovaries, cul-de-sac, and pelvic floor is on the same side as previous studies (14-17). However, the results of agreement in terms of fallopian tube endometriosis based on our study were not concordant with the abovementioned studies.

Several reasons could justify the variety of results about fallopian tube involvement. The first reason is the fallopian tube abnormalities reported in almost 30% of women with endometriosis, which may mislead the radiologist during the TVS. The second reason is due to the serosal and subserosal fluid accumulations, which lead to peritubular adhesions, fluid distention, and eventually hydrosalpinx, which could mimic tubal endometriosis. On the other hand, there may be intraluminal endometriosis or, rarely, a hydrosalpinx appearing as a cystic tubular structure, which may not be detected as a true lesion. The last reason is the presence of a non-specific hematosalpinx, which may be considered as endometriosis (18-20).

The retrospective study on 420 cases has detected the same results as ours in terms of fallopian tubes and all other anatomical lesions (20).

Several studies agree with our results in investigating bowel endometriosis by TVS and its agreement with IO findings (21-24). However, the study noticed that the depth estimation of endometriosis in different sites of the bowel is different, and TVS is not enough to aid in the rectum because of its inaccuracy in diagnosing mucosal layer infiltration. Thus, it would not help the surgeons decide whether to perform discoid or segmental resection (19, 18).

Based on our results, the agreement between TVS and IO findings in terms of bowel involvement is significant, and this indicates that TVS would be aiding in terms of bowel endometriosis. The surgeons' decision-making is multifactorial, and cannot be limited to TVS reports. Many factors such as the diameter of the infiltrating lesion, the presence of lumen stenosis, and clinical manifestations should be considered seriously.

Infiltration of endometriosis into the rectovaginal septum is clinically important to distinguish. While recto cervical endometriosis is treated with local excision or ablation, rectovaginal endometriosis treatment often requires bowel resection (25, 26). TVS is the first-line imaging modality to evaluate the DIE nodules of the rectovaginal septum, and its sensitivity is reported to be between 9-78%. This indicates that the detection of endometriosis of the rectovaginal septum is technically difficult (3). However, based on our results, its agreement with IO findings was 100%, which reflects that it is highly dependent on the experience and knowledge of the radiologist.

Finally, as described in our results, the exact detection of various DIE lesions by TVS in different locations, particularly at the site of the ovaries, bowel, Douglas pouch, and rectovaginal septum, provides the surgeons with a better preoperative view to plan their surgery. Ovaries (left-right) endometrioma had sensitivities of 82. 19-75.38% and, specificity of 75-88.5%, also 94.4-91.4%, and specificity of 0.5-0.33% for ovarian adhesions.

“It assessed the presence of OMA and adhesions (12). They reported sensitivities (84%) and specificities (95.6%) for endometrioma and sensitivities (79.6%) and specificities (91.9%) for adhesions. There was some chance of bias within their results, as they found ovarian adhesions present in 94% of women with endometrioma. Given that the 2 operators performing the scans in their study had a “high level of experience", it is possible that the operators had come to assume that adhesion may have been concurrently present with an endometrioma. Also, if 94% of women with an endometrioma have adhesions, it can safely be inferred that these will be present at the surgery without prior confirmative imaging. In the study in Iran (17), a sensitivity of 62.2% and a specificity of 95.7% were reported in the “ovarian fossa endometrioma". Although they did not define what US features, they considered suggestive of DIE in the ovarian fossa, the use of the term ovarian fossa suggests that this represented more than endometrioma. The sensitivity was reported by those (1) who were able to show 100% sensitivity for the diagnosis of OMA. Although they provided a detailed description of what they classified as endometrioma, they too were nonspecific regarding what exactly constituted ovarian disease (1).”

In this study, bowel DIE had 88.34% sensitivities and specificity of 44.44%. The sensitivities and specificity of bowel DIE were very different in other studies “because biggest challenge from a surgical perspective that was, DIE affecting the bowel presents (7, 14, 20, 27). May be a specialized colorectal surgeon required to assist the gynecologist in the removal of these lesions. Depending on the severity, segmental resection may be indicated, making the procedure highly invasive and placing a substantial burden on the person's quality of life, highlighting the importance of accurate surgical planning and individual counseling (14).”

In this study, bowel DIE had 7.1% sensitivity and, specificity of 98.87%. In the study of Albozi et al. (7). “The TVS, transrectal sonography, and magnetic resonance imaging observed a sensitivity of 100% for the detection of ureteral DIE. Another study using TVS as the first-line screening imaging technique suggested another study (10). It was unclear as to why some sensitivities were lower than others. In the normal pelvis, the uterosacral ligaments were not seen with ultrasono, which may account for some of the poor accuracies encountered in this region (10, 7).”

## 5. Conclusion

In conclusion, TVS allows a precise preoperative evaluation in planning the right surgery policy associated with the improvement in the capability of surgical techniques. This confirms that an expert radiologist can aid the surgeon in the preoperative evaluation and IO management.

##  Conflict of Interest

The authors declare that there is no conflict of interest.
